# Removal of Tape-Worm by Balsam Copaiba

**Published:** 1876-01

**Authors:** 


Removal of Tape-Worm by Balsam of Copaiba.—At a recent meeting of the New York Pathological Society, Dr. Salvatore Caro presented a tape-worm, and recited the following interesting history of its removal by balsam of copaiba, after the failure of other agents. The patient was a man who had suffered from tape-worm for years, and had tried, with but little benefit, koosso, pumpkin-seed, kamela, oil of turpentine, and male-fern. He had also a gleet of long standing, and for the treatment of this disease came under observation.

Dr. Caro was not aware, at that time, that he suffered from tape-worm, and he ordered, for the cure of the gleet, teaspoonful doses of balsam of copaiba every two hours till it acted on the bowels, and, after that, to diminish the quantity. After the administration of the first dose the bowels moved freely, and in the stool there was found the whole of the tape-worm. Dr. Caro said that he was not aware of the remedy being administered to patients suffering from tape-worm, and had obtained the result by mere accident.

Dr. Briddon said that he had been usually successful with the ethereal extract of the oil of male-fern. His method of treating cases was to give a black draught twenty-four hours before exhibiting the remedy, and in the interim to feed the patient on beef-tea. Twelve hours before giving the male-fern he gave a dose of castor-oil. The preparation of male-fern that he used was the ethereal extract prepared by Merck, of Vienna. He had administered the fluid extract of male-fern, but had obtained no benefit from it.

